# Anatomic Characteristics Associated with Head Splitting in Cabbage (*Brassica oleracea* var. *capitata* L.)

**DOI:** 10.1371/journal.pone.0142202

**Published:** 2015-11-04

**Authors:** Wenxing Pang, Yoon-Young Kim, Xiaonan Li, Su Ryun Choi, Yunbo Wang, Chang-keun Sung, Subin Im, Nirala Ramchiary, Guangsheng Zhou, Yong Pyo Lim

**Affiliations:** 1 Molecular Genetics and Genomics Lab, Department of Horticulture, Chungnam National University, Daejeon, Republic of Korea; 2 College of Horticulture, Shenyang Agricultural University, Shenyang, China P.R.; 3 Department of Food Science and Technology, College of Agriculture and Biotechnology, Chungnam National University, Daejeon, Republic of Korea; 4 Translational and Evolutionary Genomics Lab, School of Life Sciences, Jawaharlal Nehru University, New Delhi, India; 5 College of Plant Science and Technology, Huazhong Agricultural University, Wuhan, China P.R.; Huazhong university of Science and Technology, CHINA

## Abstract

Cabbage belonging to Brassicaceae family is one of the most important vegetables cultivated worldwide. The economically important part of cabbage crop is head, formed by leaves which may be of splitting and non-splitting types. Cabbage varieties showing head splitting causes huge loss to the farmers and therefore finding the molecular and structural basis of splitting types would be helpful to breeders. To determine which anatomical characteristics were related to head-splitting in cabbage, we analyzed two contrasting cabbage lines and their offspring using a field emission scanning electron microscope. The inbred line “747” is an early head-splitting type, while the inbred line “748” is a head-splitting-resistant type. The petiole cells of “747” seems to be larger than those of “748” at maturity; however, there was no significant difference in petiole cell size at both pre-heading and maturity stages. The lower epidermis cells of “747” were larger than those of “748” at the pre-heading and maturity stages. “747” had thinner epidermis cell wall than “748” at maturity stage, however, there was no difference of the epidermis cell wall thickness in the two lines at the pre-heading stage. The head-splitting plants in the F_1_ and F_2_ population inherited the larger cell size and thinner cell walls of epidermis cells in the petiole. In the petiole cell walls of “747” and the F_1_ and F_2_ plants that formed splitting heads, the cellulose microfibrils were loose and had separated from each other. These findings verified that anomalous cellulose microfibrils, larger cell size and thinner-walled epidermis cells are important genetic factors that make cabbage heads prone to splitting.

## Introduction

Cabbage (*Brassica oleracea* var. *capitata* L.) is one of the many varieties of *Brassica oleracea*, which is in the Brassicaceae family. Brassicas are important to humankind for their economic, nutritional, and potential anticancer value. The economic value of cabbages is reduced when their heads split or crack, and when they are attacked by insects.

The cracking/splitting trait has been a topic of interest for breeders and researchers for a long time. This trait has been observed in many fruits and vegetables such as cherry, apple, litchi, tomato, and Chinese cabbage [1–5]. The polygenic inheritance of cabbage head-splitting was reported as early as 1972 [6]. In tomato, the characteristics of large fruit size, low skin tensile strength, and thin skin were shown to be related to susceptibility to cracking [4]. It is believed that irregular overhead watering is one of the factors causing splitting or cracking, as well as sub-optimal humidity, temperature, and light conditions [4]. The thickness of the cuticular membrane was shown to be strongly related to cracking in cherry tomato [7].

Expansins are proteins that loosen the cell wall by weakening the noncovalent network formed by cellulose, hemicellulose, and pectins [8]. Expansins have functions in plant cell growth, cell separation, cell wall loosening, and cell wall disassembly [9–11]. All of these processes have been shown to contribute to fruit cracking in apple and litchi [3,12]. The cellulose microfibril patterns and arrangement when cells divide, expand, and differentiate in inflorescences and leaves of *Arabidopsis thaliana* have been observed by field emission scanning electron microscopy (FESEM) [8]. The results of those studies showed that the microstructure of plant cells was closely related to the cracking or splitting of fruits and vegetables.

Although cabbages susceptible to head-splitting were found, no detail study at molecular or at anatomical structure has been done. Previous study in splitting type cabbage genotypes at maturity stage reported to observe a large leaf mesophyll cells with a loose structure [13]. Furthermore, in our previous preliminary study, we found thinner cell walls, lower cell density, larger cell size, and anomalous cell wall structure in head splitting line at heading stage in cabbage [14]. However, the anatomical characteristics related to the head-splitting trait and their inheritance in cabbage have not been studied in detail. The aim of this study, therefore, was to verify the anatomical characteristics related to head splitting by observing two diverse cabbage inbred lines, and to evaluate the genetic inheritance of head-splitting in their offspring.

## Materials and Methods

### Plant Materials

**Fig 1:**
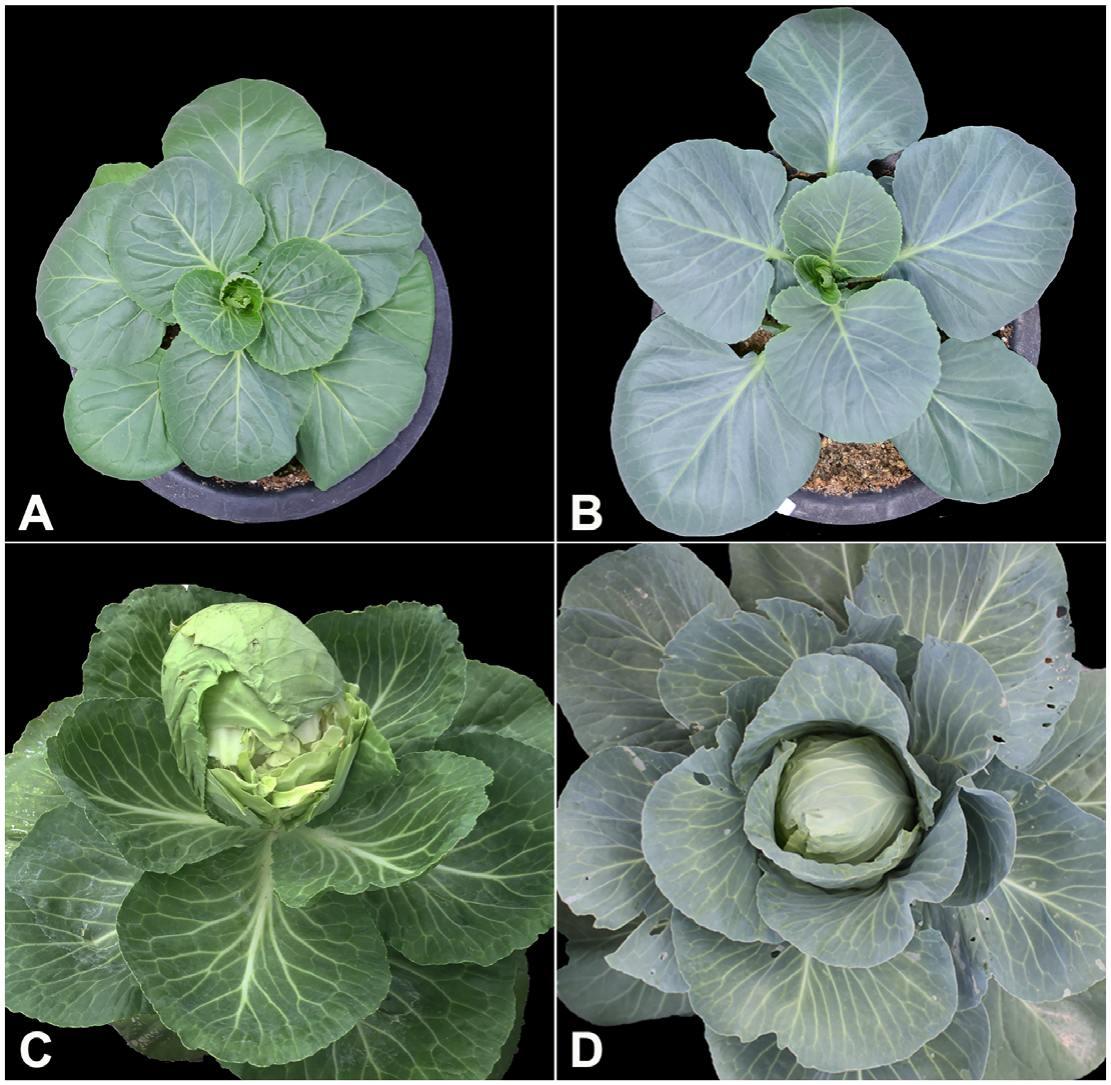
Phenotype of “747” and “748” at young and mature stages. (A) “747” at young stage; (B) “748” at young stage; (C) “747” at mature stage; (D) “748” at mature stage. The images are not indicative of the actual size of the plants.

The inbred line “747” is an early head-splitting line, while the inbred line “748” is a head-splitting-resistant type (Fig 1). Details of these lines are provided elsewhere [14]. Briefly, “747” and “748” have a growth period of approximately 100–120 days from germination to maturity. An F_2_ population was obtained from bud self-pollination of a single hybrid F_1_, which was derived by crossing “747” and “748”. The two diverse cabbage inbred lines and the F_1_ and F_2_ population were used to observe anatomical characteristics by FESEM. Thirty seeds of each parental line with F_1_ and F_2_ were germinated at the 21^st^ February 2014 in 40-well multipots. After one month, the seedlings were transferred to 25-cm diameter pots. Plants were watered carefully onto the soil directly to avoid any possible contamination or damage to the epidermal wax [15]. Leaf samples were collected from plants at different growth stages. All of the plants were grown in glasshouse at Chungnam National University, Daejeon, South Korea.

### Field Emission Scanning Electron Microscopy

The anatomical characteristics of different leaf samples were observed under a FESEM (Model: JSM-7000F; JEOL, Germany). Leaf and petiole specimens were collected from individuals of “747”, “748”, and the F_1_ and F_2_ plants at the pre-heading and maturity stages using a single edge blade. At the pre-heading stage, the fourth leaf from the terminal bud was collected from 2-month-old seedlings of “747” and “748”. At maturity, the outer leaf of the head was collected from mature plants of the parental line, F_1_ and F_2_ plants of head-splitting and non-splitting types. At least three to five biological repeats were taken for observing anatomical characteristics from each sample. Two different methods were used to prepare samples for observations of epidermal wax and cellular structure. To observe the epidermal wax structure, fresh leaf samples were cut into small pieces, quickly frozen using liquid nitrogen, and then freeze-dried (Laboratory Freeze Dryer, Model: 7.001.300.950, Denmark) for 24 h. For cellular structure observations, we used the method described in our previous study [14]. Fresh sample sections were fixed in 70% ethanol and then dehydrated in an ethanol series. The tissues were dehydrated in an isoamyl acetate: ethanol series and critical-point dried (CPD; Model: CPD 030 critical point dryer, Bal-Tec, Germany) with CO_2_. All specimens were sputter-coated with platinum for 100–150 s and photographed under the FESEM at 10–20 kV.

## Results

### Cellular Structure Observed in Petiole Cross-Sections

**Fig 2:**
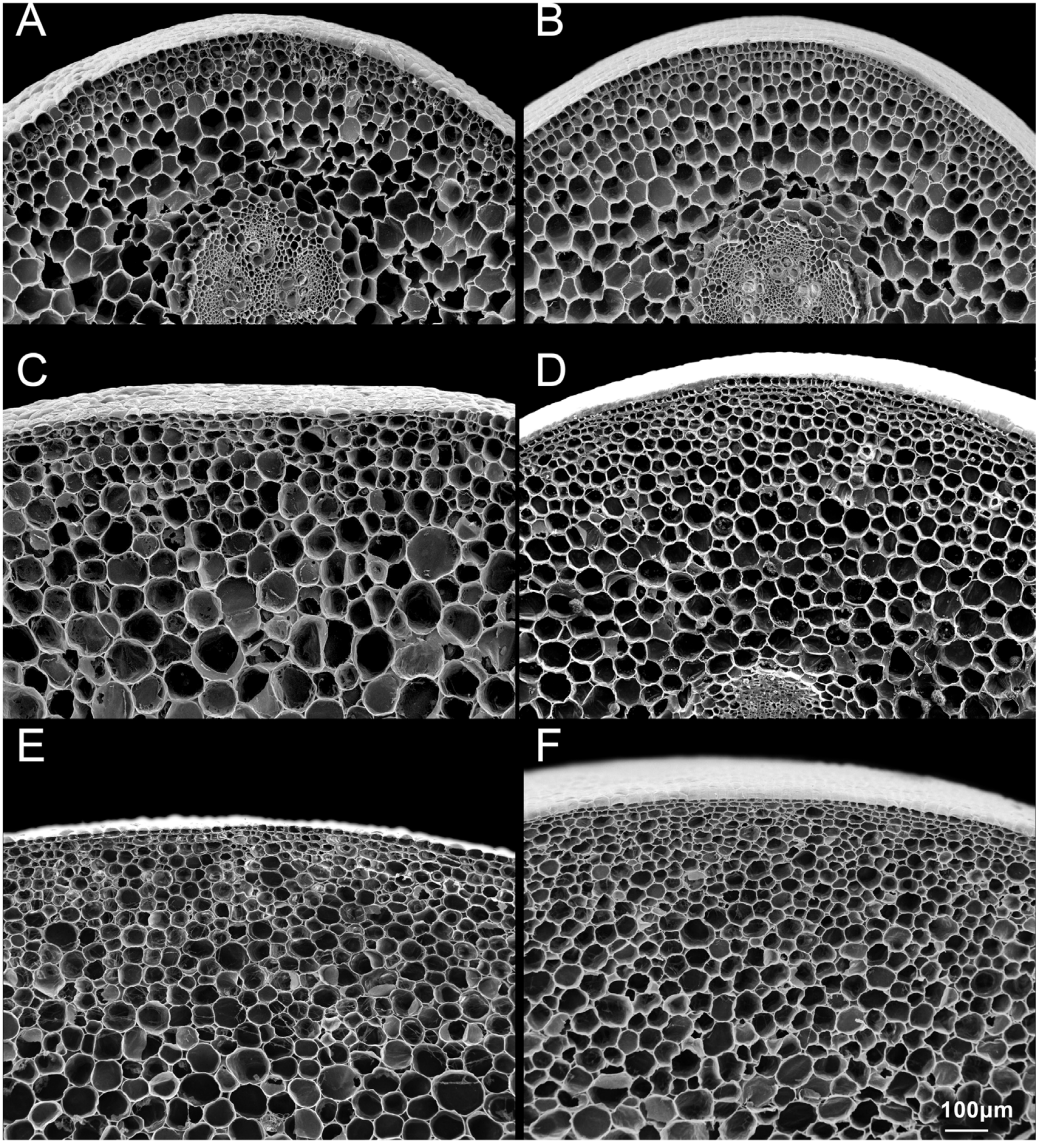
Cell structure in petiole cross-sections of *Brassica oleracea* var. *capitata* L. at pre-heading and maturity stages. Pre-heading stage of “747” (A) and “748” (B); mature “747” (C) and “748” (D); head-splitting individuals from F_1_ (E) and F_2_ population (F) at maturity.

**Fig 3:**
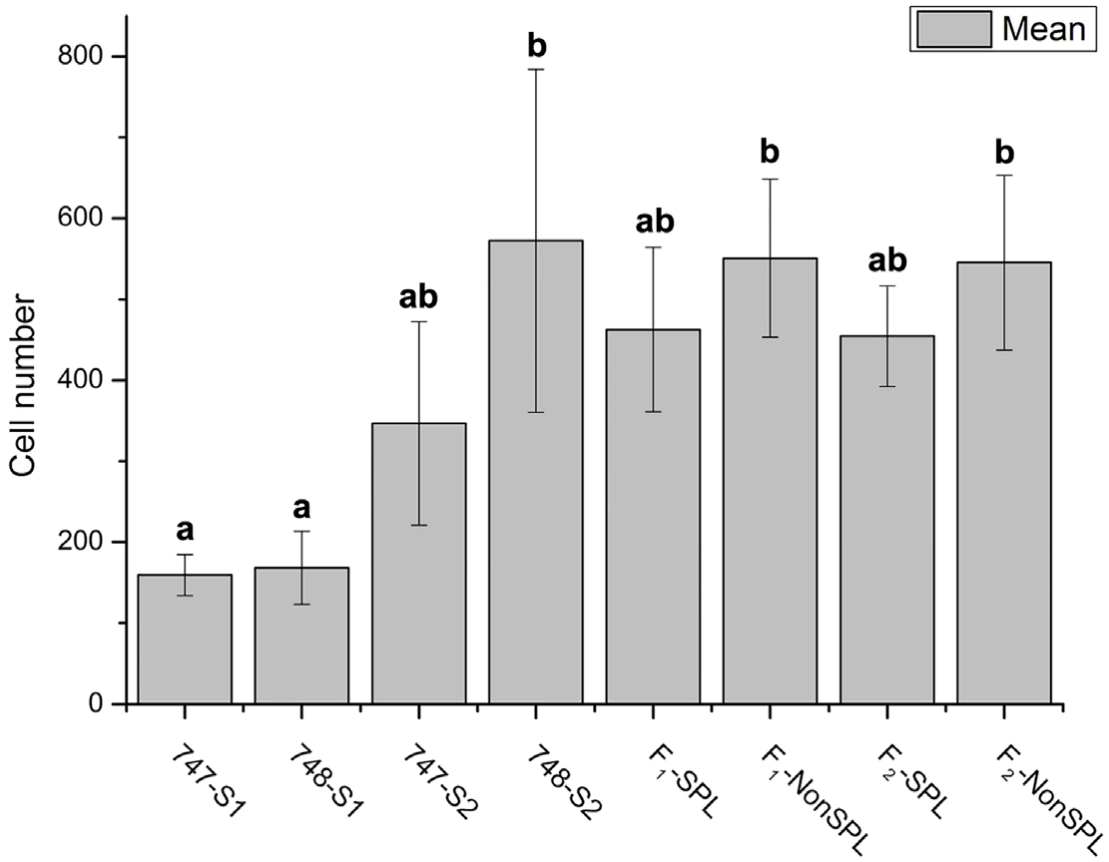
Cell number (number/mm^2^) in petiole cross-section of *Brassica oleracea* var. *capitata* L. and their comparison among different groups of plants. Different letters indicate a significant difference between groups. (p < 0.05; one-way ANOVA and Tukey's multiple comparison tests). “747-S1” and “748-S1” indicate “747” and “748” at pre-heading stage; “747-S2” and “748-S2” indicate “747” and “748” at maturity; “F_1_-SPL” and “F_2_-SPL” indicate F_1_ and F_2_ head-splitting individuals; “F_1_-NonSPL” and “F_2_-NonSPL”, F_1_ and F_2_ non-head-splitting individuals.

Since the thickness of cell walls and cell size were reported that related to head splitting at heading stage in cabbage [14]. We tried to confirm it by comparing the cell size between head splitting and non-head splitting individuals at different growing stages. The petiole cell size was observed from parental lines and their offspring at pre-heading and mature stages. The petiole cell size showed no significant different between “747” and “748” at the pre-heading stage. However, the petiole cells of “747” were larger, and less dense, than those of “748” at maturity. (Figs 2 and 3). The cell numbers (number of cells per mm^2^) of “747” and “748” were not significantly different at the pre-heading and maturity stages, and there was no significant difference in cell numbers between head-splitting and non-head-splitting individuals in the F_1_ and F_2_ population (Fig 3). In both “747” and “748”, the cell numbers were higher at maturity than at the pre-heading stage.

### Epidermis Cell Structure

**Fig 4:**
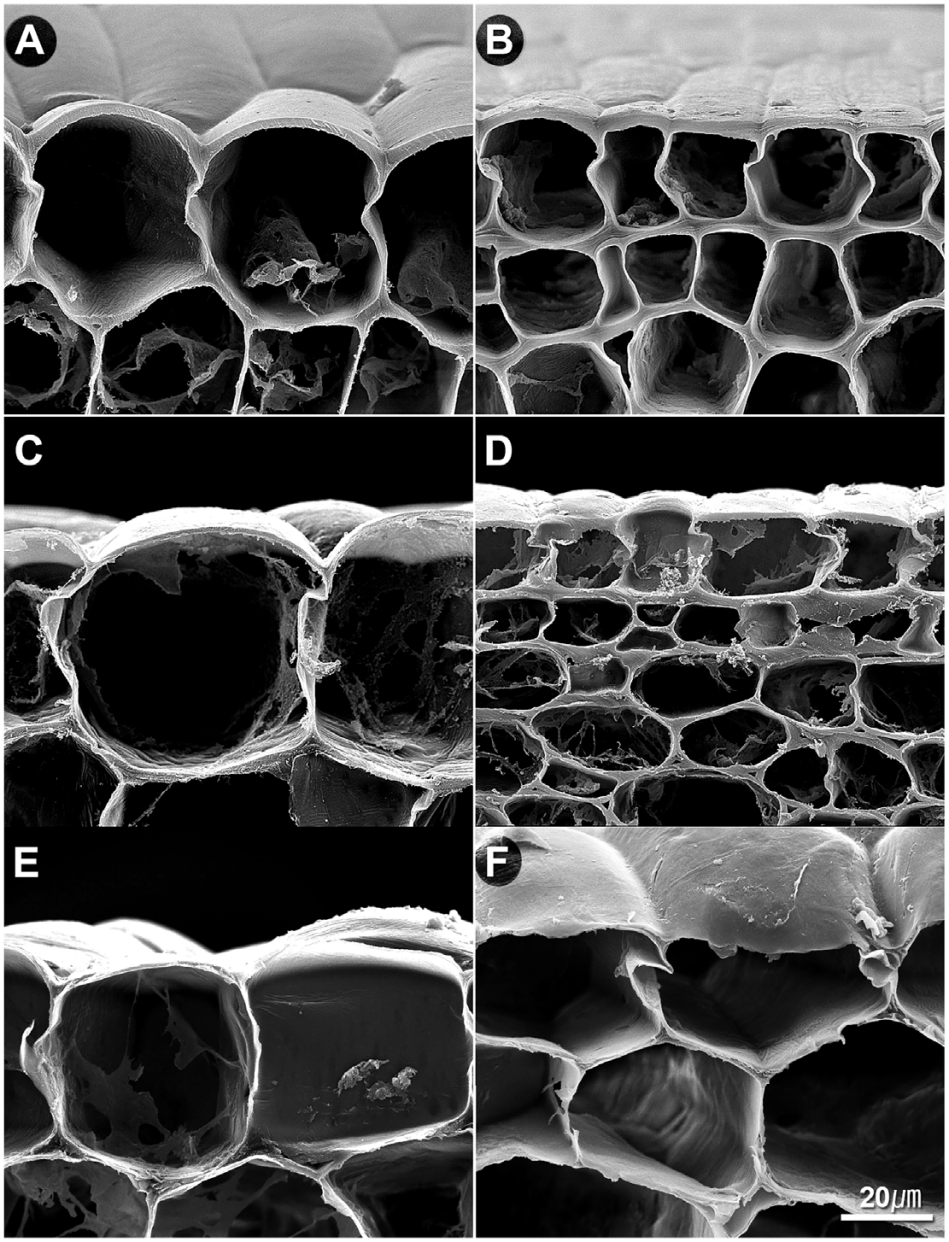
Petiole epidermis cell size and cell wall thickness in *Brassica oleracea* var. *capitata* L. at pre-heading and maturity stages. Pre-heading stage of “747” (A) and “748” (B); mature stage of “747” (C) and “748” (D); head-splitting plants of F_1_ (E) and F_2_ (F) at maturity.

**Fig 5:**
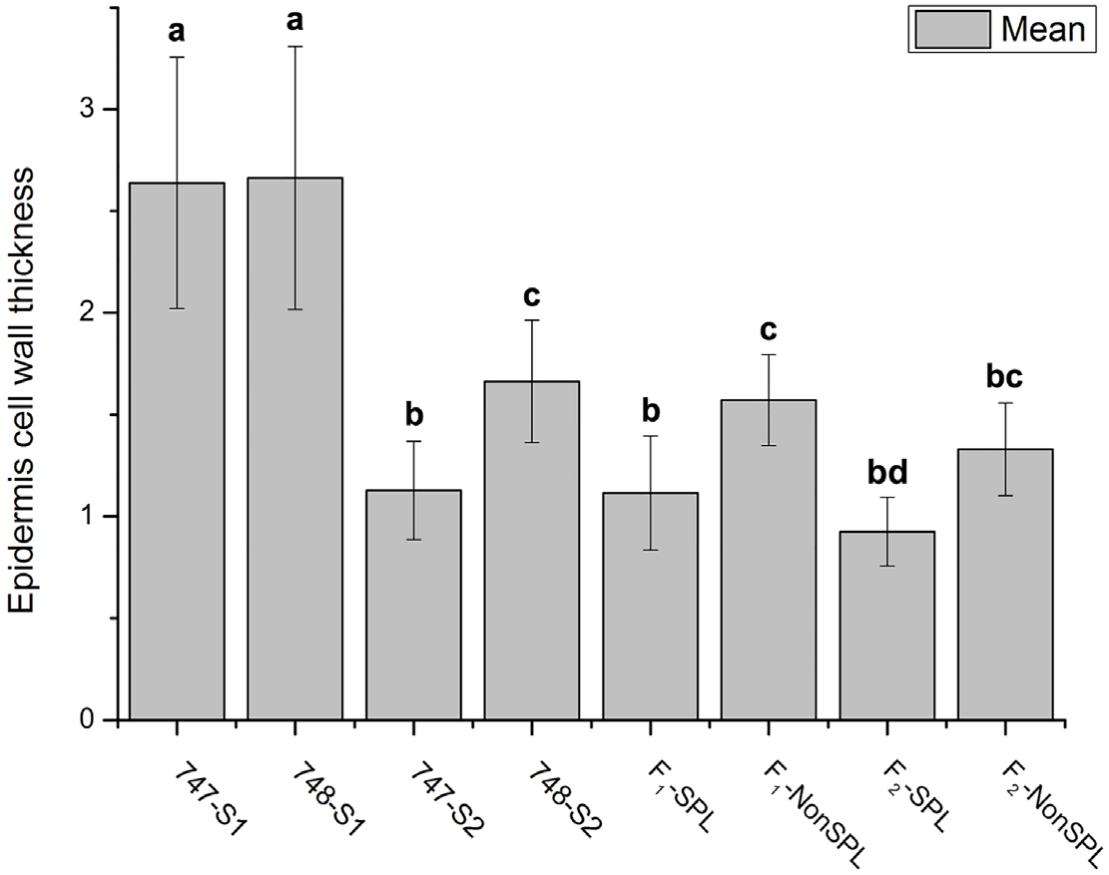
Epidermal cell wall thickness in petiole cross-section of *Brassica oleracea* var. *capitata* L. and their comparison among different groups of plants. Different letters indicate a significant difference between groups. (p < 0.05; one-way ANOVA and Tukey's multiple comparison tests). “747-S1” and “748-S1” indicate “747” and “748” at pre-heading stage; “747-S2” and “748-S2” indicate “747” and “748” at maturity; “F_1_-SPL” and “F_2_-SPL” indicate F_1_ and F_2_ head-splitting individuals; “F_1_-NonSPL” and “F_2_-NonSPL”, F_1_ and F_2_ non-head-splitting individuals.

We also tried to compare the epidermis cell structures between head splitting and non-head splitting individuals flowing the growing stages. Epidermis cell structures were observed in “747” and “748” at the pre-heading and maturity stages, and in head-splitting and non-head-splitting plants from the F_1_ and F_2_ population at the maturity stage (Figs 4 and 5; S1 and S2 Figs). The lower epidermis cells and hypodermis cells of “747” were larger than those of “748” at the pre-heading and maturity stages (Fig 4). The lower epidermis cell walls were significantly thinner in “747” than in “748” at maturity (Fig 5). Compared with non-head-splitting individuals, head-splitting individuals had thinner lower epidermis cell walls (Fig 5). The epidermal cell wall thickness differed significantly between head-splitting and non-head-splitting individuals at maturity (Fig 5). Taken together, the head-splitting plants had larger lower epidermis cells and hypodermis cells, as well as thinner lower epidermis cell walls than non-head-splitting plants.

### Hypodermis Cell Wall Structure

**Fig 6:**
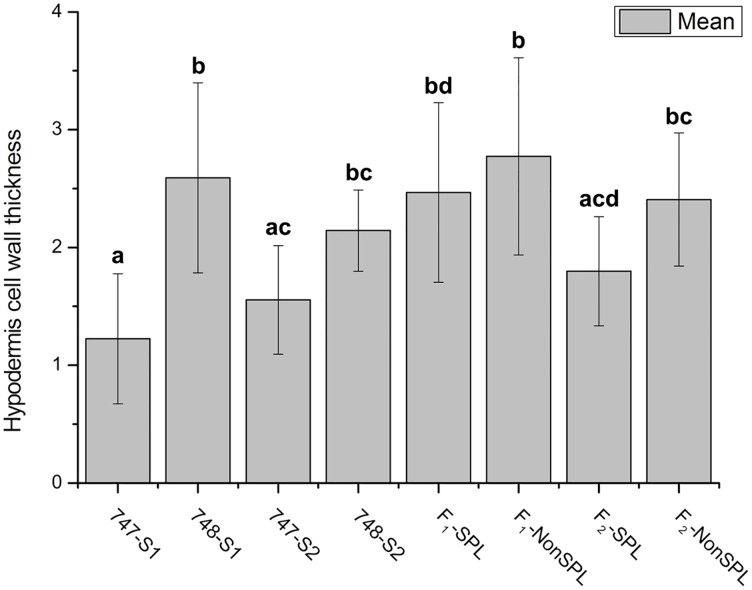
Hypodermis cell wall thickness in petiole cross-section of *Brassica oleracea* var. *capitata* L. and their comparison among different groups of plants. Different letters indicate a significant difference between groups. (p < 0.05; one-way ANOVA and Tukey's multiple comparison tests). “747-S1” and “748-S1” indicate “747” and “748” at pre-heading stage; “747-S2” and “748-S2” indicate “747” and “748” at maturity; “F_1_-SPL” and “F_2_-SPL” indicate F_1_ and F_2_ head-splitting individuals; “F_1_-NonSPL” and “F_2_-NonSPL”, F_1_ and F_2_ non-head-splitting individuals.

**Fig 7:**
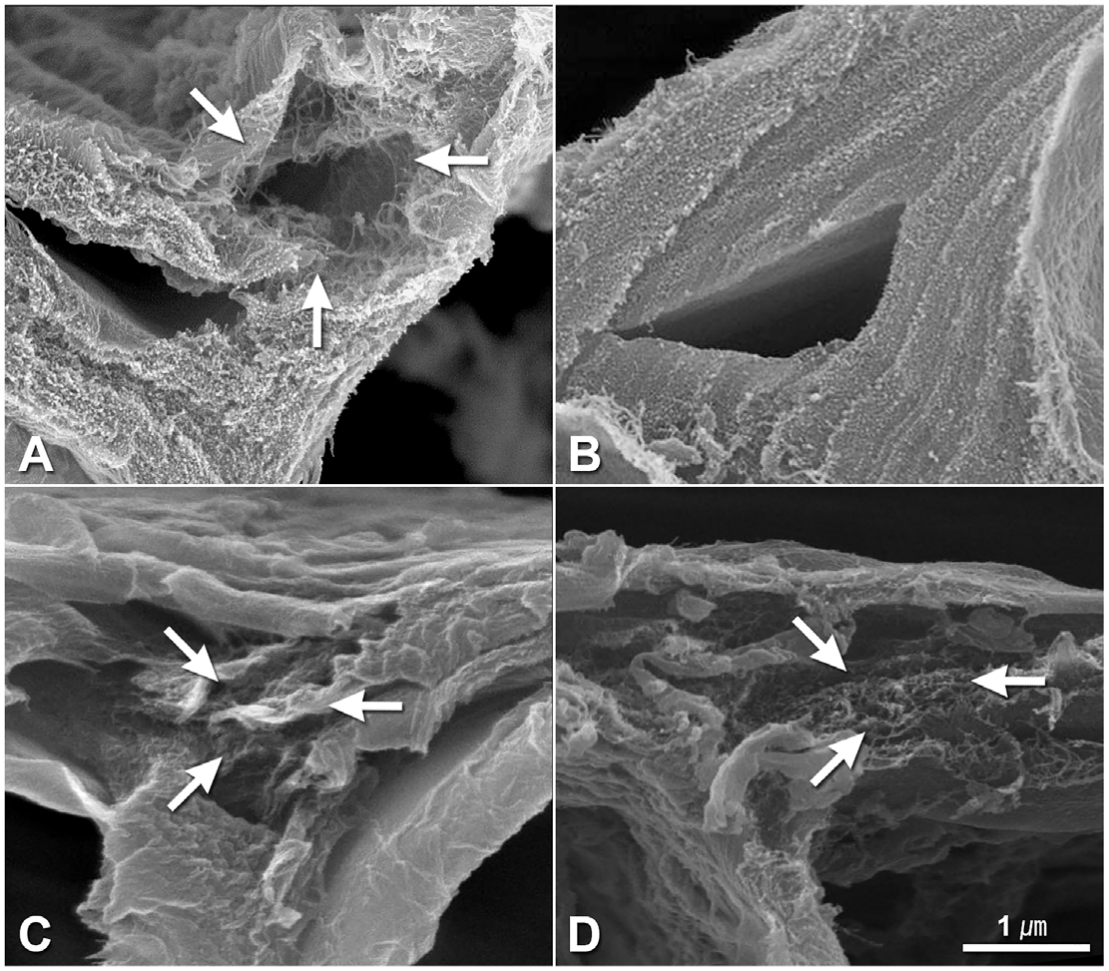
Hypodermis cell wall structure in petiole cross-section of *Brassica oleracea* var. *capitata* L. at maturity. Cell wall structure of “747” (A) and “748” (B). Cell wall structure of head-splitting plants from F_1_ (C) and F_2_ (D) population. White arrows indicate anomalous cell wall structure.

The cell wall ultrastructure of parental lines and their offspring were analyzed using FESEM in this study. The cell walls of hypodermis cells of “747” were significantly thinner than those of “748” at the pre-heading stage (Fig 6). However, there was no significant difference in the thickness of hypodermal cell walls between parental lines and between head-splitting and non-head-splitting individuals of F_1_ and F_2_ at maturity (Fig 6). An anomalous cell wall structure was observed in “747” and in head-splitting individuals of F_1_ and F_2_ at maturity, but not in “748” or non-head-splitting individuals of F_1_ and F_2_ (Fig 7). In the head-splitting individuals, the cellulose microfibrils were loose and had separated from each other (Fig 7A, 7C and 7D). In contrast, the cellulose microfibrils of non-head-splitting individuals were closely linked together (Fig 7B).

### Epicuticular Wax Pattern

**Fig 8:**
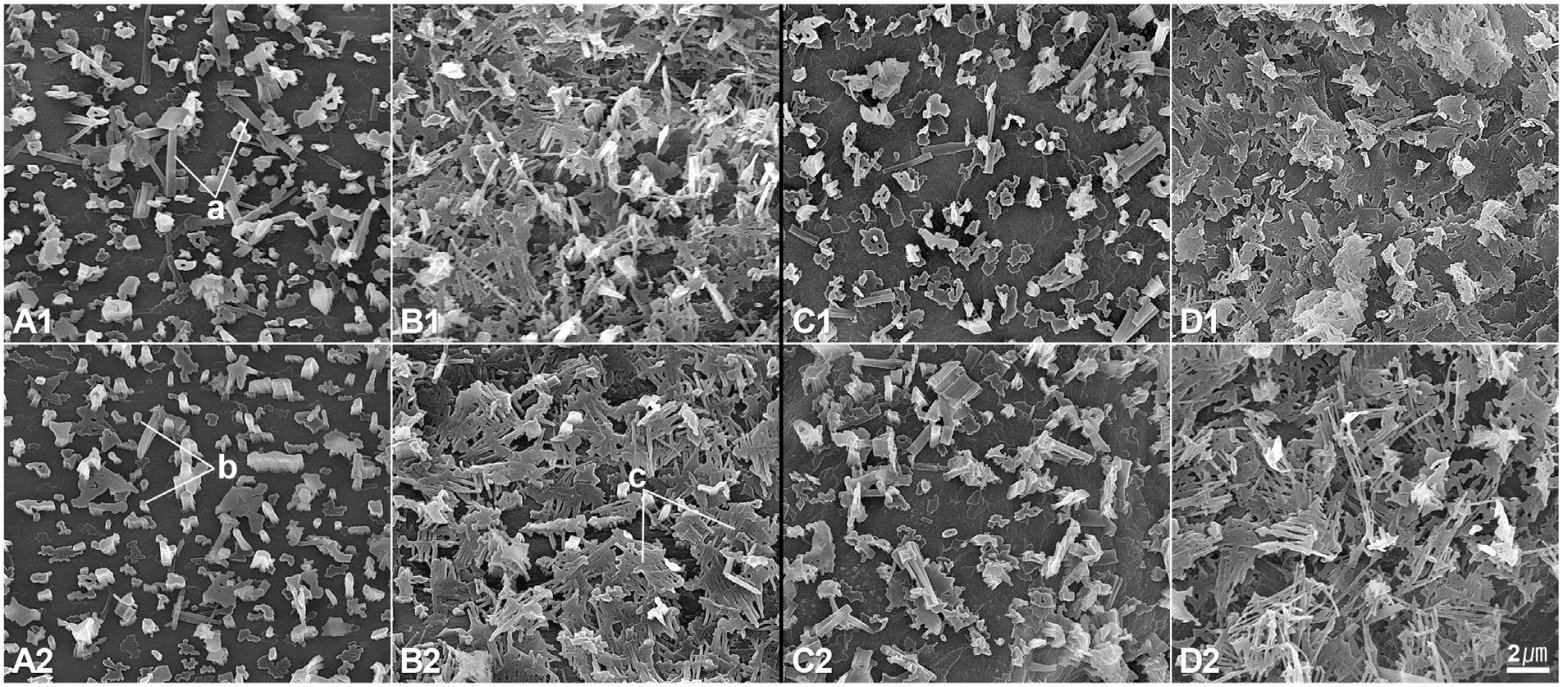
Wax patterns of *Brassica oleracea* var. *capitata* L. “747” and “748” at pre-heading stage. (A) Petiole epidermis of “747” (1: adaxial; 2: abaxial). (B) Petiole epidermis of “748” (1: adaxial; 2: abaxial). (C) Leaf epidermis of “747” (1: adaxial; 2: abaxial). (D) Leaf epidermis of “748” (1: adaxial; 2: abaxial). Wax type: a, long columnar type; b, short columnar type; c, joint-plate type.

**Fig 9:**
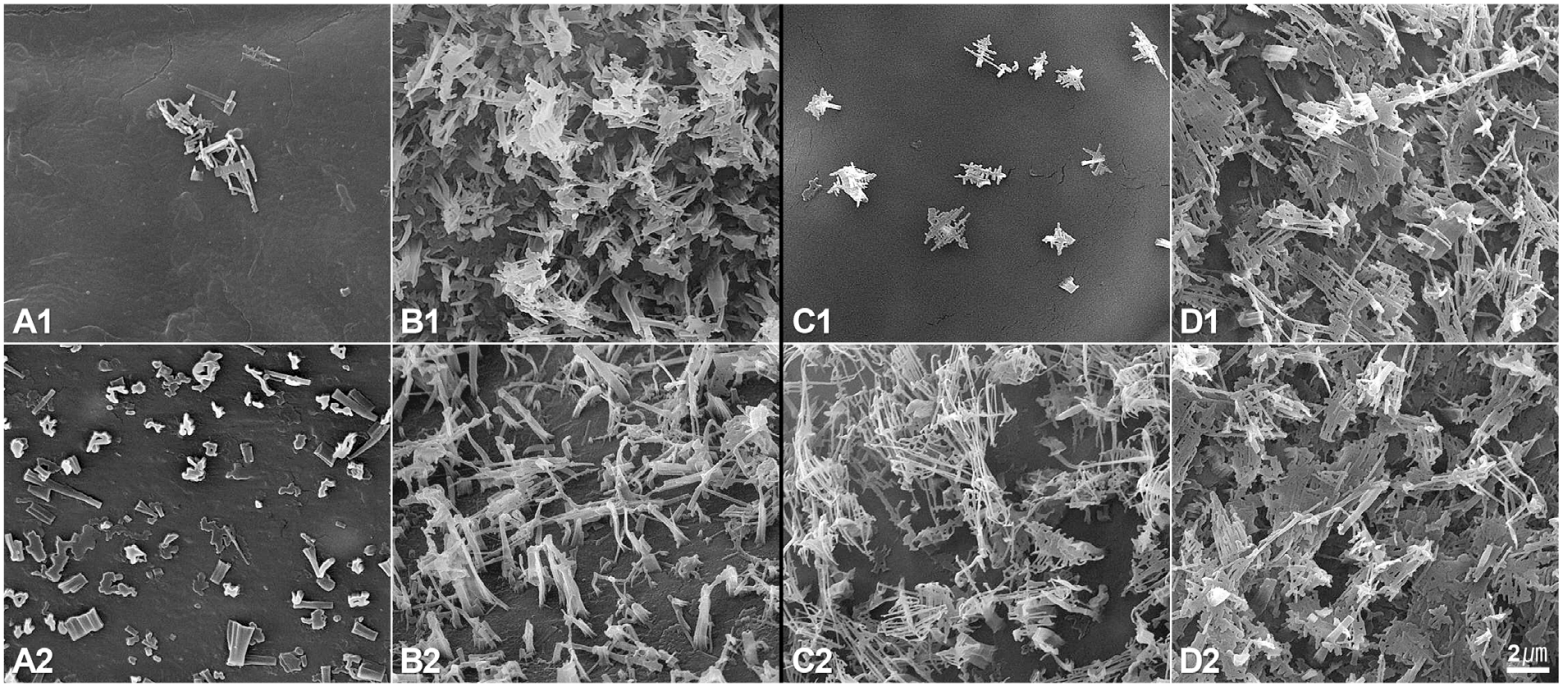
Wax patterns of *Brassica oleracea* var. *capitata* L. “747” and “748” at maturity. (A) Petiole epidermis of “747” (1: adaxial; 2: abaxial). (B) Petiole epidermis of “748” (1: adaxial; 2: abaxial). (C) Leaf epidermis of “747” (1: adaxial; 2: abaxial). (D) Leaf epidermis of “748” (1: adaxial; 2: abaxial).

The amount of epicuticular wax on leaves of “748” were significantly different from “747” which can be distinguished easily by visual observation. Under FESEM, three types of wax were detected in the parental lines “747” and “748” i.e. short columnar-type, long columnar-type, and joint-plate-type (Fig 8). In both the leaf and petiole, there was less epicuticular wax on “747” than on “748” at the pre-heading and maturity stages (Figs 8 and 9). The upper epidermis wax layer on the leaf and petiole was smaller than the lower epidermis wax layer in “747” at maturity. However, there was no significant difference in the size of the wax layer between the upper epidermis and lower epidermis on the leaf and petiole of “747” at the pre-heading stage (Fig 8A1, 8A2, 8C1 and 8C2; Fig 9A1, 9A2, 9C1 and 9C2). In “748”, there was no difference in the size of the wax layer between the upper epidermis and lower epidermis on the leaf and petiole at the pre-heading and maturity stages (Fig 8B1, 8B2, 8D1 and 8D2; Fig 9B1, 9B2, 9D1 and 9D2). The wax layer on the epidermis of the leaf and petiole was thicker and denser in “748” than in “747”, although there was a substantial wax layer on the lower epidermis of the leaf and petiole of “747” (Figs 8 and 9).

## Discussion

Cracking or splitting leads to serious economic losses in many fruit and vegetable crops including cherry, apple, litchi, tomato, Chinese cabbage, and cabbage [1–6,16]. Cabbage is one of the most important vegetables, and is cultivated worldwide. In the present study, the anatomic characteristics of two diverse cabbage inbred lines and their offspring at different growth stages were analyzed by FESEM to identify factors related to head splitting and their inheritance.

Larger fruits are believed to be more susceptible to cracking. However, in cabbage, plants with a comparatively small head were found to be susceptible to head-splitting [14]. Thus, the head size was shown to have no relationship with head-splitting in this study. Observations of head-splitting showed that it usually started from the petiole at maturity. Therefore, the microstructure of the petiole was systematically analyzed. Although the cells of the head-splitting-susceptible line “747” seems to be larger than those of the splitting-resistant line “748” at maturity, however, no difference in cell size between the two lines at the pre-heading and maturity stages was detected in the ANOVA. Moreover there was no significant difference in petiole cell size between head-splitting and non-splitting individuals in the F_1_ and F_2_ population. However, lower epidermis cells were larger, and their cell walls were thinner, in “747” than in “748” at the pre-heading and maturity stages. The same difference in anatomical characteristics was observed between head-splitting and non-splitting plants in the F_1_ and F_2_ population, indicating that the head-splitting offspring inherited the larger cell size and thinner cell wall of lower epidermis cells from “747”.

A previous study on head-splitting showed that at maturity, head-splitting-susceptible cabbage leaves contained fewer layers of mesophyll cells, and the mesophyll cell layer had a looser structure and larger cellular spaces [13]. Smaller cells result in a higher density of cell walls, providing greater rigidity and strength to tissues. Therefore, smaller cells were considered to be more resistant to bucking and breaking [17]. In the present study we also observed the same. Larger cells can take up more water than smaller cells, and thinner cell walls rupture more easily than thicker cell walls. Therefore, the larger cell volume and thinner cell wall of lower epidermis cells may be important genetic factors in susceptibility to head splitting in cabbage (Figs 4 and 5). The mean thickness of hypodermis cell wall was not significantly different between head-splitting and non-head-splitting plants, indicating that hypodermis cell wall thickness may not be a factor in head splitting.

Previous studies have shown that fruit and vegetable cracking or splitting is mainly affected by environmental and genetic factors [4]. The environmental factors include irrigation, temperature, light, and humidity. The genetic factors related to cracking or splitting include plant architecture, fruit size, cell structure (cell number, size, and density, and cell wall thickness), cuticle thickness, and secondary metabolites content [4,18]. In the present study, anomalous cellulose microfibrils were observed in the petiole cell wall of “747” and in the F_1_ and F_2_ plants that formed split heads. The cellulose microfibrils were loose and had separated from each other (Fig 7). This trait was inherited in the F_1_ and F_2_ plants. Therefore, anomalous cellulose microfibril structure is another genetic factor related to head splitting in cabbage.

All of the factors contributing to head splitting combine to form weak points in the petiole of head-splitting plants. Head splitting occurs as a result of these weak points as a short-board effect, even though head-splitting plants have small cells and thick-walled lower epidermis cells in some parts of the petiole (S1 and S2 Figs). Expansins have been shown to function in plant cell growth, cell wall loosening, cell separation, and cell wall disassembly [11,19]. Expansin genes associated with fruit cracking have been found in apple and litchi [3,8]. Our anatomic results suggested that expansin genes involved in cabbage head splitting and we speculate that aberrant expression of expansin genes led to the loosening and separation of cellulose microfibrils, ultimately leading to head splitting. Polygenic inheritance of the head-splitting trait in cabbage was also reported in 1972. Moreover, six quantitative trait loci (QTLs) conferring resistance to head splitting have been detected on chromosomes 2, 4, and 6 [6,14]. The F_1_ individuals shown diversity on head splitting resistance with an average resistance value of 0.42 under uniform experimental conditions which indicated that it was controlled by quantitative trait loci [14].

In the present study, large cell size, low cell density, thin cell walls of lower epidermis cells, and anomalous cell wall structure were verified in all head-splitting plants in the F_1_ and F_2_ population. This result indicated that these characteristics are important genetic factors contributing to cabbage head-splitting, a trait that is inherited stably from parents to offspring.

Epicuticular wax on leaves plays an important role in defense. We observed that there was a thicker epicuticular wax layer on “748” than on “747” on both the petiole and the leaf at all growth stages. In particular, the upper epidermis wax layer on the leaf and petiole of “747” was much thinner at maturity than at the pre-heading stage. This may be because the upper epidermis of the leaf and petiole was wrapped inside the head at maturity. However, the lower epidermis wax layer on the leaf and petiole of “748” was not significantly reduced at maturity by analyzing the images, the wax contents need to be quantitative analyzed further. The epicuticular waxes of plants are complex mixtures of long-chain acyl lipids. Plants secrete waxes on the surface of their cuticles as a way to reduce water loss, resist insect attack, and prevent infection by pathogenic fungi and bacteria [20]. There was no evidence that epicuticular waxes were related to head splitting in cabbage. Previous studies have reported that glossy-leaf cabbage was resistant to diamondback moth (DBM) and that the epicuticular wax content was related to the degree of damage by cabbage pests [21–23]. Eight QTLs were mapped for DBM resistance using the same parental lines, and the waxy-leaf phenotype was found to be more resistant to DBM in the F_2/3_ segregating population [24]. These results suggest that epicuticular waxes are more strongly related to DBM resistance than to head-splitting.

The results of this study demonstrate that several factors contribute to cabbage head-splitting; the large cell size and thin cell wall of lower epidermis cells, and the anomalous structure of cellulose microfibrils that form a weak point. Our findings suggest that the thickness of the hypodermis cell wall and epicuticular wax layer are not associated with head splitting in cabbage. In future research, the roles of expansin genes and epidermis cell wall development-related genes in head splitting should be studied in more detail.

## Supporting Information

S1 Fig Petiole cell structure in a head-splitting individual from F_1_plants. (A) Petiole cross-section; (B) enlargement of weak point from (A); (C) enlargement of normal cell structure from (A).
(TIF)

S2 Fig Petiole cell structure in a head-splitting individual from F_2_ population. (A) Petiole cross-section; (B) enlargement of weak point from (A); (C) enlargement of normal cell structure from (A).
(TIF)
