# Supplementary figures and images for: Anatomic Characteristics Associated with Head Splitting in Cabbage (Brassica oleracea var. capitata L.)
Source: PLoS One. 2015 Nov 4;10(11):e0142202. doi: 10.1371/journal.pone.0142202 (PMC4633201; doi:10.1371/journal.pone.0142202)

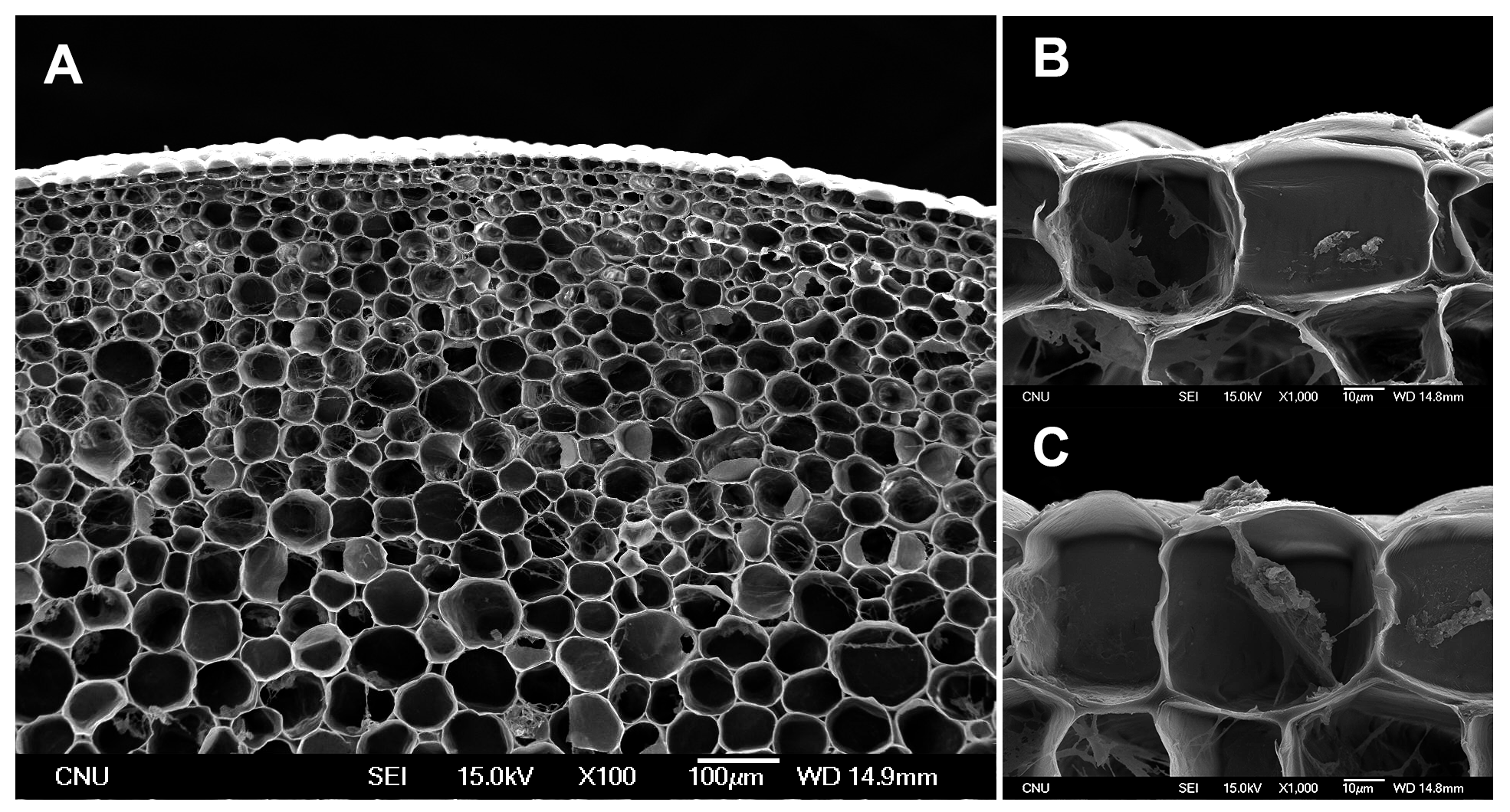

Supplement: S1 Fig — (A) Petiole cross-section; (B) enlargement of weak point from (A); (C) enlargement of normal cell structure from (A). (TIF) [file pone.0142202.s001.tif]

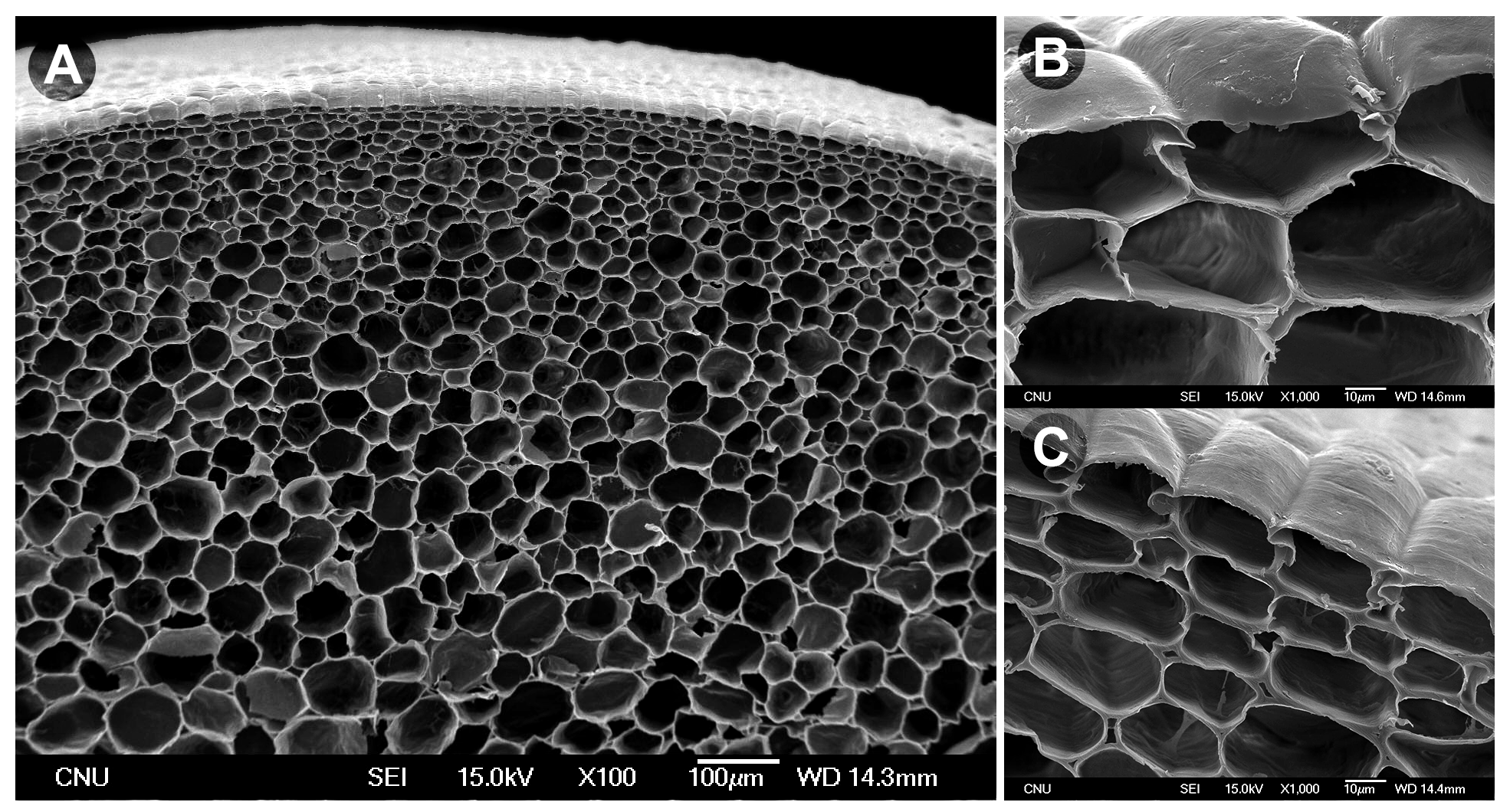

Supplement: S2 Fig — (A) Petiole cross-section; (B) enlargement of weak point from (A); (C) enlargement of normal cell structure from (A). (TIF) [file pone.0142202.s002.tif]
